# cGAS-STING pathway mediation in osteoclast function and bone fracture healing

**DOI:** 10.3389/fimmu.2026.1789655

**Published:** 2026-05-21

**Authors:** Miaomiao Zheng, Li Zhao, Shensheng Nian, Qiang Wei, Panyu Zhou, Hao Tang

**Affiliations:** 1 Department of Orthopedics, Changhai Hospital, Naval Military Medical University, Shanghai, China; 2 Department of Orthopedics, General Hospital of Southern Theater Command of PLA, Guangzhou, Guangdong, China

**Keywords:** cGAS, fracture, healing, osteoclast, STING

## Abstract

**Objective:**

The cGAS-STING pathway serves as a key mediator of inflammation. The aim of this study is to explore the biological role and molecular mechanisms of the cGAS-STING pathway in the fracture healing process, with a particular focus on its function during the early inflammatory phase.

**Methods:**

A murine femoral fracture model was utilized to investigate the activation of the cGAS-STING pathway during the early and late stages of bone healing. The methodologies encompassed transcriptome sequencing and immunohistochemistry. Pathway modulation was accomplished through the application of the STING inhibitor H-151 and the activator SR-717, with the effects being assessed via *in vivo* experiments (transcriptome sequencing, microCT, safranin O-fast green staining, and TRAP staining) and *in vitro* assays (TRAP staining, F-actin ring formation, bone resorption tests, qPCR, and Western blot analysis).

**Results:**

Transcriptome analysis revealed a higher expression of STING on day 7 compared to day 21. Immunohistochemistry results also demonstrated significant activation of the cGAS-STING pathway within the fracture callus, particularly during the initial stages of healing. Transcriptome sequencing indicated that the activation of the cGAS-STING pathway by SR-717, in contrast to H-151, predominantly impacted osteoclasts while activating the NF-κB pathway. Results from microCT, safranin O-fast green, and TRAP staining suggested that the activation of the cGAS-STING pathway contributed to facilitating fracture healing and osteoclastogenesis. *In vitro* experiments further confirmed that SR-717 enhanced osteoclast formation and activity, while H-151 had an inhibitory effect. The underlying molecular mechanisms further demonstrated that the activation of the cGAS-STING pathway enhanced the transmission of the RANKL-induced NF-κB signaling pathway.

**Conclusion:**

The cGAS-STING pathway played a significant role in the early stages of healing in murine femoral fractures, accelerating the process of fracture repair. This pathway primarily influenced osteoclast differentiation and was associated with the key pathway of osteoclast formation, NF-κB.

## 1 Introduction

Fractures are a common type of traumatic injury that not only impact individual health but also have a significant effect on global public health (1). The healing process of fractures generally involves three stages: inflammation, repair, and remodeling (2), with the inflammatory phase being particularly crucial as it marks the initiation of the body’s immune response and the beginning of fracture repair (3). Osteoclasts play a vital role in this stage, as they are responsible for removing damaged bone tissue and breaking down the bone matrix, thus providing space for new bone formation (4). While the importance of osteoclasts during the inflammatory phase of bone healing is well-recognized, their specific regulatory mechanisms require further investigation and exploration.

In recent years, the cGAS-STING pathway has garnered widespread attention as a significant mediator of inflammation (5). cGAS, a cytoplasmic DNA sensor, forms an active dimer in the presence of cytosolic DNA, catalyzing the synthesis of the second messenger molecule cGAMP from ATP and GTP. cGAMP is considered the natural ligand for the STING protein, activating STING and facilitating its transition from the endoplasmic reticulum to its active form (6, 7). STING, a transmembrane protein located in the endoplasmic reticulum, oligomerizes into tetramers in the ER-Golgi intermediate compartment and the Golgi apparatus upon activation (8). Through the action of adaptor proteins, STING recruits and activates TBK1 (TANK-binding kinase 1), leading to the phosphorylation of IRF3 (Interferon Regulatory Factor 3). Phosphorylated IRF3 then enters the nucleus, activating the transcription of interferon genes. Simultaneously, the STING signaling pathway can also activate the transcription factor NF-κB, inducing the participation of various inflammatory mediators in the inflammatory response (9, 10).

Following a fracture, tissue damage and vascular rupture trigger an inflammatory response, leading to the infiltration of inflammatory cells and the release of inflammatory mediators (11). The activation and regulation of these cells and mediators facilitate the involvement of other cell types in the repair process, such as osteoclasts, osteoblasts, and endothelial cells (12, 13). Osteoclasts, in particular, release acidic substances and enzymes to degrade bone, clearing damaged bone tissue and making space for new bone formation (14). Active osteoclasts also release a variety of growth factors and cytokines, such as RANKL, which regulate the migration and proliferation of osteoblasts (15). These processes are largely regulated by the Nuclear Factor κB (NF-κB) signaling pathway. Activation of the NF-κB pathway in osteoclasts can regulate the expression of a series of genes related to osteoclast function, including those related to osteoclastogenesis, function, and survival (16). Therefore, there is a close relationship between the cGAS-STING pathway and osteoclasts. The activation of the cGAS-STING pathway may promote the activation of NF-κB, playing a crucial role in the formation and activation of osteoclasts, and thus participating in regulating the fracture healing process.

Although the cGAS-STING pathway is known to regulate osteoclastogenesis in inflammatory bone diseases, its temporal activation and functional significance during the early inflammatory phase of fracture healing remain unexplored. In this study, we investigate the spatiotemporal dynamics of cGAS-STING activation in a murine femoral fracture model and demonstrate its pro-healing role via promoting osteoclast differentiation through NF-κB signaling, thereby providing new insights into the stage-specific regulation of fracture repair.

## 2 Materials and methods

### 2.1 Animals

Wild-type (WT) C57BL/6J mice (6–8 weeks old) from the Naval Medical University Animal Laboratory were housed in a specific pathogen-free (SPF) animal facility with a controlled humidity of 50 ± 5%, temperature of 23 ± 1 °C, and a 12-hour light/dark cycle (lights on at 6:00 AM). Adequate food and water were provided. All animal experiments were ethically approved by the Shanghai Changhai Hospital (the First Affiliated Hospital of Naval University of the Chinese Navy) Animal Ethics Committee and were conducted in accordance with the committee’s guidelines.

### 2.2 Mouse fracture experiments and drug administration

Male C57BL/6J mice (6–8 weeks old) were anesthetized with pentobarbital, the midshaft of the left femur was exposed and cut with a steel rod, and a needle was inserted into the marrow cavity for fixation, followed by suturing the incision with 4–0 silk thread. After preparing the fracture model, mice were randomly divided into three groups. Drug interventions began the day following surgery; for the inhibitor group, 200μl of STING inhibitor H-151 solution (750nmol) (Selleck, USA) was administered daily via intraperitoneal injection. For the activator group, 200μl of STING activator SR-717 solution (30mg/kg) (MCE, USA) was administered daily via intraperitoneal injection. A control group received an equivalent volume of PBS buffer as a control, with injections continuing for one week.

### 2.3 Micro-CT

Two and four weeks post-surgery, mouse femurs were fixed in 4% paraformaldehyde for 24 hours. The specimens were then positioned on a Micro-CT platform for scanning, with parameters such as bone volume (BV), bone volume fraction (BV/TV), and bone mineral density (BMD) evaluated and analyzed.

### 2.4 Histological analysis

Firstly, femur specimens were fixed with 4% paraformaldehyde, followed by decalcification with 15% EDTA. The treated specimens were then dehydrated and embedded in paraffin, and the paraffin blocks were sectioned into 5-10μm thick slices. These slices were then deparaffinized with ethanol of appropriate concentrations and stained for STING immunohistochemistry, safranin O-fast green staining, and TRAP staining for tissue analysis.

### 2.5 RNA-seq analysis

Total RNA was extracted from collected callus tissue for mRNA-seq analysis. Sequencing and analysis of all samples were completed by Gene Denovo Biotechnology Co., Ltd in Guangzhou.

### 2.6 Extraction of mouse bone marrow macrophages

Bone marrow-derived macrophages (BMMs) were obtained from the femurs and tibias of 6-week-old male C57BL/6J mice. These cells were cultured in α-MEM culture medium containing 1% penicillin/streptomycin and 10% fetal bovine serum, supplemented with 20 ng/mL M-CSF, in a 37 °C, 5% CO2 incubator. After four days of culture, when confluence reached about 90%, the adherent cells were considered bone marrow mononuclear macrophages. Cells were then harvested using trypsin digestion for further experimental research.

### 2.7 Cell viability assay

The harvested BMMs were seeded at a density of about 10^4 cells/well in 96-well plates with 25 ng/ml M-CSF for 24 h. After attachment, the medium was replaced with various concentrations of H-151/SR-717 solution (0, 0.25, 0.5, 1, 2, 5, 10, or 20 μM) and incubated for another 24 h. Then, 10 μL of CCK-8 solution was added to each well and incubated in a 37 °C, 5% CO2 humidified environment for 4 hours. Absorbance was measured at 450 nm using a Synergy H1 (BioTek, Winooski, VT, USA).

### 2.8 Osteoclast differentiation and TRAP staining

The harvested BMMs were seeded at a density of about 10^5 cells/well in 48-well plates with 20 ng/ml M-CSF and 50 ng/ml RANKL in α-MEM medium. Additionally, 5μm H-151 solution, 2μm SR-717 solution, and an equivalent volume of DMSO were added as controls, with the medium changed every 2 days. After 5–7 days, once fused cells were observable under a light microscope, cells were fixed with 4% paraformaldehyde for 20 minutes. Staining was then performed according to the TRAP staining kit instructions for 30 minutes, followed by three PBS washes. Finally, cells were observed under a light microscope, and the number and area of TRAP-positive osteoclasts containing at least three nuclei were quantified using ImageJ software.

### 2.9 F-actin ring immunofluorescence

Following the intervention described above, and once fused cells were observable under the light microscope, F-actin ring staining was performed. Cells were first fixed with 4% paraformaldehyde at room temperature for 20 minutes, then permeabilized with 0.15% Triton X-100 for 5 minutes, and washed three times with PBS. Phalloidin staining solution was prepared and added to the cells, which were then incubated in the dark for 30 minutes. Subsequently, DAPI staining solution was applied to cover the cells for nuclear staining for 3 minutes. Observations were made under a fluorescence microscope, and statistical analysis was conducted using ImageJ software.

### 2.10 Bone resorption assay

Sterilized bovine cortical bone slices were placed in 48-well plates, and the harvested BMMs were seeded onto the 48-well plates and bone slices as described above, and cultured for 5–7 days. When osteoclast formation was observed in wells without bone slices, the culture was continued for an additional 48 hours. After this period, cells on the surface of the bovine bone slices were washed off, and the bone slices were collected. Bone resorption pits on the bovine bone slices were observed under a scanning electron microscope, and quantitative analysis was performed using ImageJ software.

### 2.11 Quantitative polymerase chain reaction

BMMs were seeded in 12-well plates at a density of 4×10^5 cells per well and treated as described above for 24 hours. Total RNA was extracted from the BMMs using an RNA extraction kit (R401-01, Vazyme) and reverse-transcribed into cDNA using a reverse transcription kit (11141EZ60, YESEN). Real-time quantitative PCR was then performed using a SYBR Green PCR kit (11202ES03, YESEN) on a QuantStudio 5 (Applied Biosystems) system. GAPDH was used as the reference gene. The cycle threshold (Ct) values for target genes were collected, and relative mRNA expression levels were calculated using the 2^-ΔΔCt method. The primer sequences used were:

### 2.12 WB

The intervention method was consistent with the previously described procedures, followed by the extraction of total cellular proteins for Western blot analysis. Briefly, cells were washed thrice in PBS and lysed in lysis buffer containing protease and phosphatase inhibitors. The protein concentration was determined using a BCA Protein Assay Kit. Samples were separated by sodium dodecyl sulfate-polyacrylamide gel electrophoresis (SDS-PAGE) and transferred onto PVDF membranes. The membranes were blocked with 5% bovine serum albumin at room temperature for 1 hour, followed by three washes with TBST. Incubation with primary antibodies against STING, phosphorylated STING (p-STING), NF-κB p65, phosphorylated NF-κB p65 (p-NF-κB p65), CTSK, cFOS, and ACTIN was performed overnight at 4 °C. The next day, after washing the membranes thrice with TBST, they were incubated with the corresponding secondary antibodies at room temperature for 2 hours. Following three washes with TBST, chemiluminescence was detected using the Bio-Rad imaging system (Bio-Rad, USA) with an enhanced chemiluminescence (ECL, Santa, USA) method, and images were analyzed using Image J software.

### 2.13 Statistical analysis

Image analysis was conducted using Image J software, focusing on selected regions for analysis. Statistical analysis was performed using GraphPad Prism version 9.0. Quantitative data were presented as mean ± standard deviation based on at least three independent experiments. Comparisons between two unpaired groups, assuming normal distribution and homogeneity of variances, were conducted using an unpaired t-test. A p-value of less than 0.05 was considered statistically significant.

## 3 Result

### 3.1 Involvement of the cGAS-STING pathway in fracture healing

**Figure 1:**
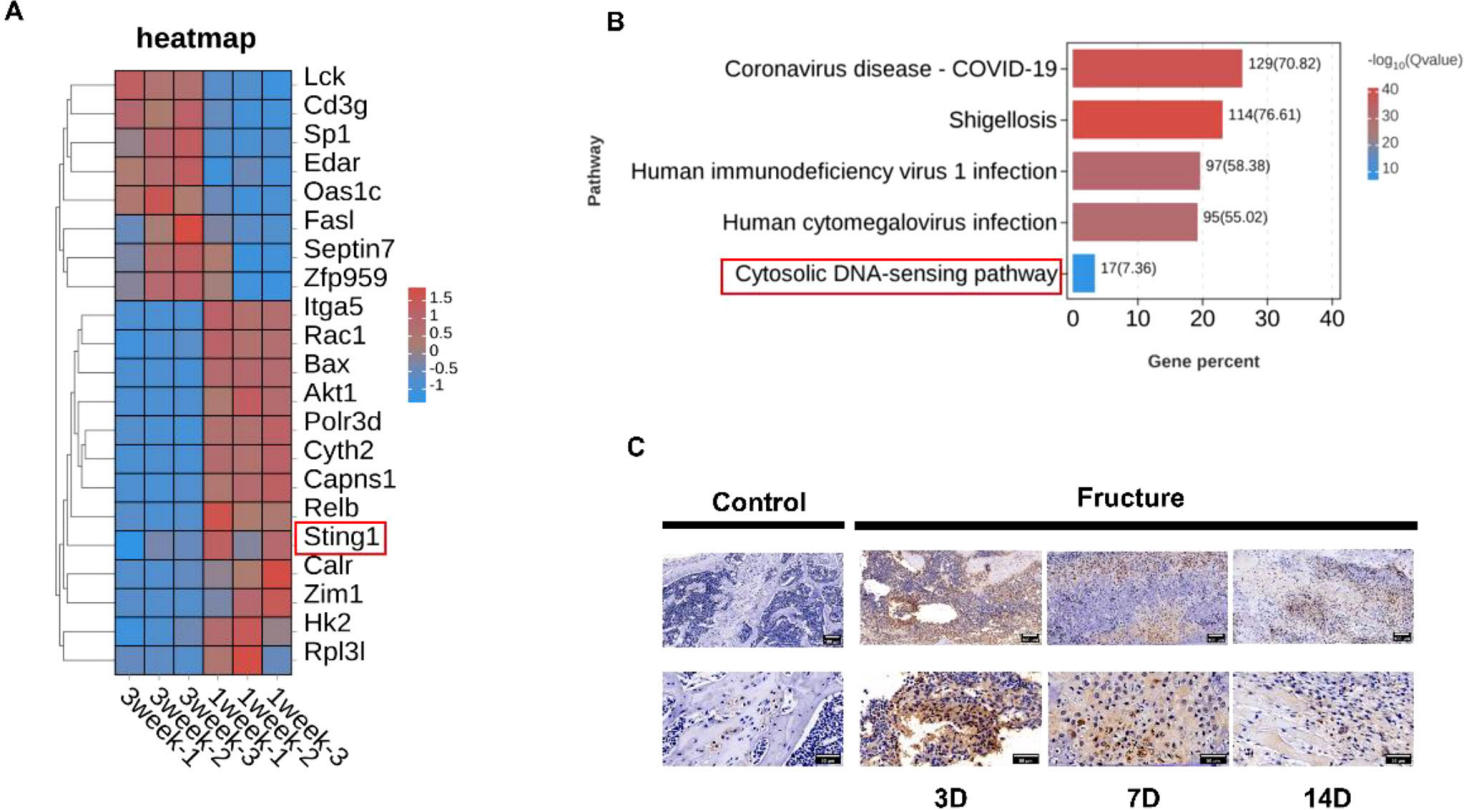
Participation of the cGAS-STING pathway in the fracture healing process. **(A)** Gene sequencing analysis. **(B)** KEGG analysis of genes related to the cGAS-STING pathway. **(C)** Representative images of STING immunohistochemical staining in mouse callus tissue on days 3, 7, and 14 post-surgery compared to the control group, with a scale bar of 100 μm.

To explore whether the cGAS-STING pathway participates in the fracture healing process in mice, we selected 6-8-week-old C57BL/6J mice and established a femoral fracture model. Given that mouse femoral fractures typically require 3–4 weeks to heal, we opted to perform transcriptomic sequencing on calluses on day 7 (early healing stage) and day 21 (later healing stage) post-surgery. Heatmap and KEGG analysis results indicated a significantly higher expression of STING in callus tissue on day 7 post-operation compared to day 21 (**Figures 1A, B**). To further determine whether the cGAS-STING pathway plays a role in the early stage of fracture healing, we evaluated the expression of STING in bone tissue on days 3, 7, and 14 post-surgery. Immunohistochemical staining revealed that STING expression was barely detectable in normal bone tissue, but was markedly upregulated following fracture, particularly at day 3 and day 7, with a gradual decline thereafter (**Figure 1C**). These results suggest that the cGAS-STING pathway is transiently activated during the early inflammatory phase of fracture healing.

### 3.2 Osteoclasts are the primary cells where the cGAS-STING pathway is activated during early fracture healing

**Figure 2:**
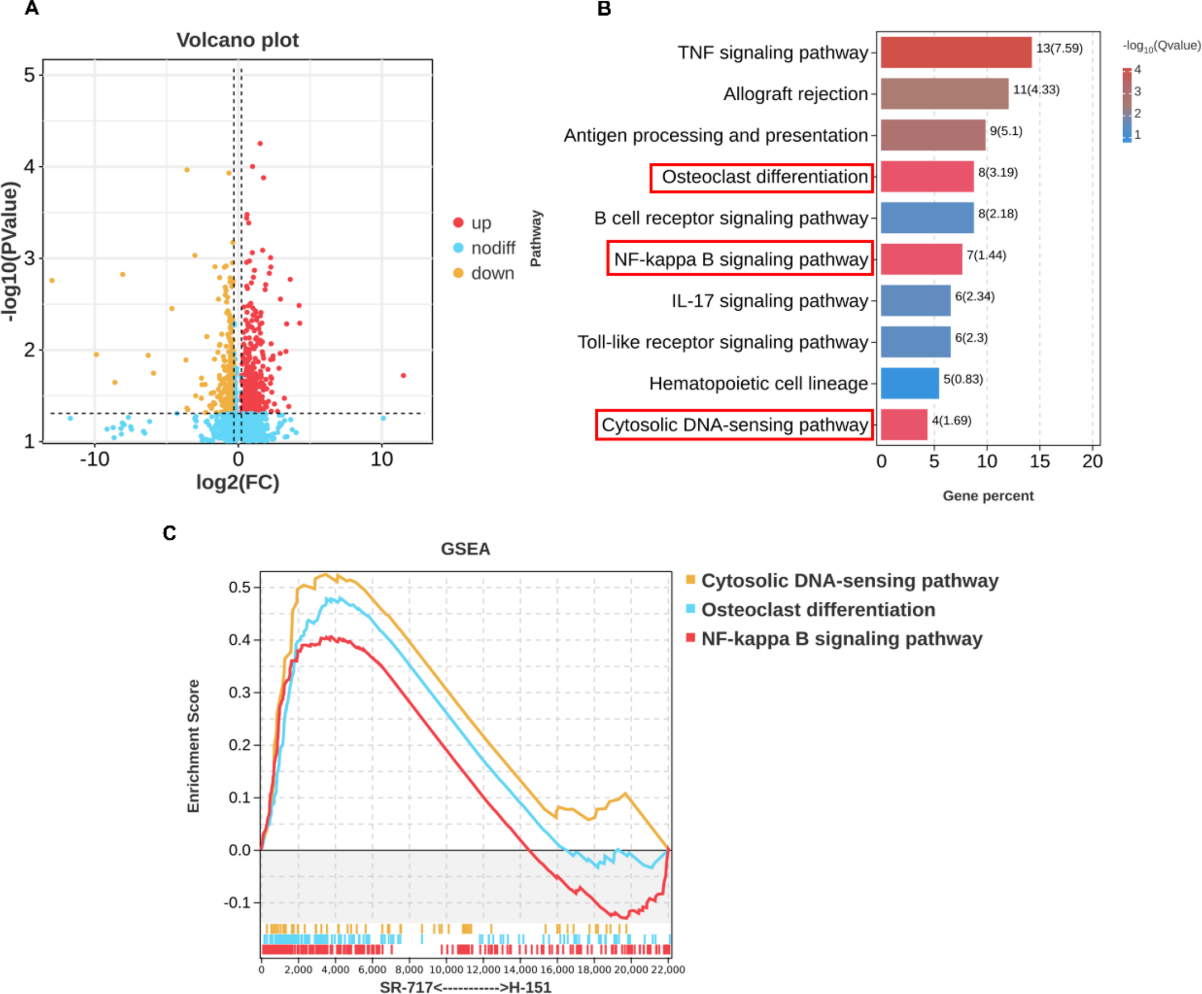
Sequencing confirmed that activation of the cGAS-STING pathway promoted osteoclast differentiation. **(A)** Volcano plot: Representation of differentially expressed genes (DEGs) in the H-151 and SR-717 groups. Downregulated DEGs are shown in yellow, and upregulated DEGs are shown in red. **(B)** KEGG analysis of genes upregulated during the activation of the cGAS-STING pathway. **(C)** GSEA analysis of specific pathways when the cGAS-STING pathway was activated.

To gain deeper insight into the regulatory role of the cGAS-STING pathway during the early stages of fracture healing, transcriptomic analysis was conducted on day 7 post-surgery on samples treated with the STING inhibitor H-151 and the STING agonist SR-717, utilizing the DESeq method to identify differentially expressed genes. Comparing both groups, a total of differentially expressed genes were identified, with 228 genes showing increased expression and 120 genes showing decreased expression (all significant at P < 0.05, with an absolute fold change of ≥1.5) (**Figure 2A**). KEGG pathway enrichment analysis revealed that, compared to other cells, the differentiation pathway of osteoclasts was significantly affected and closely associated with the NF-κB signaling pathway (**Figure 2B**). Further GSEA analysis confirmed that activation of the cGAS-STING pathway promoted the differentiation of osteoclasts and was associated with the activation of the NF-κB signaling pathway (**Figure 2C**).

### 3.3 Activation of the cGAS-STING pathway promotes fracture healing in mice

**Figure 3:**
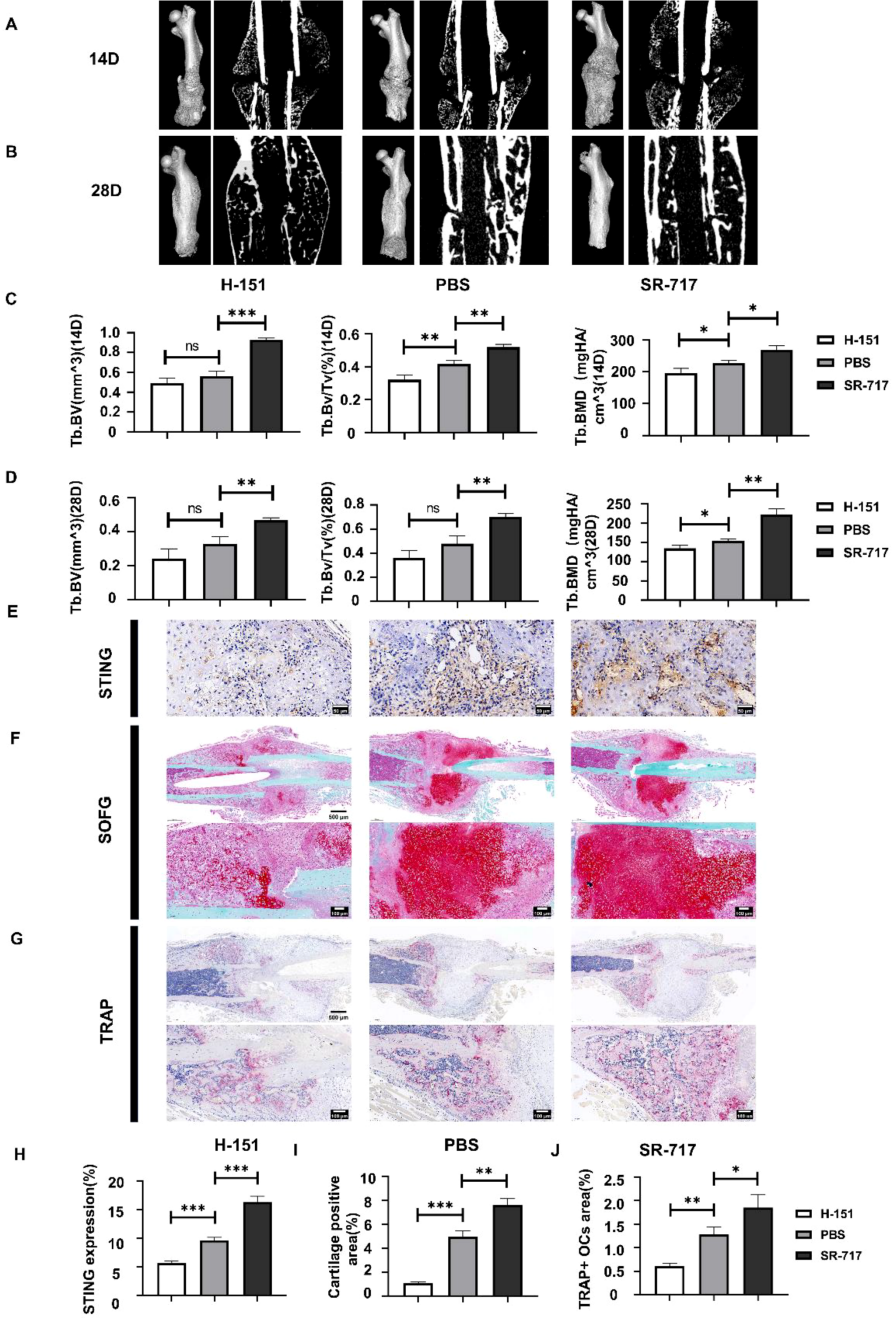
Activation of the cGAS-STING pathway promoted fracture healing in mice. **(A, B)** Micro-CT analysis results of femur samples from three groups of mice two and four weeks post-surgery: H-151, PBS, and SR-717. **(C, D)** Quantitative analysis of bone volume (Tb.BV), bone volume/tissue volume (Tb.BV/TV), and bone mineral density (Tb.BMD). **(E–G)** Representative images of immunohistochemical staining for STING **(E)**, Safranin O-Fast Green staining for cartilage **(F)**, and TRAP staining for osteoclasts **(G)** in femoral specimens from the three treatment groups (H-151, PBS control, SR-717) at 14 days post-fracture. Scale bars: 100 μm. **(H–J)** Quantitative analysis of STING-positive area **(H)**, cartilage area **(I)**, and TRAP-positive osteoclast number per bone surface **(J)**. n = 3 per group. Statistical significance: *P < 0.05, **P < 0.01, ***P < 0.001. ns, non-significant.

Fourteen and twenty-eight days post-fracture modeling, the impact of the cGAS-STING pathway on fracture healing was assessed using Micro-CT. Results indicated that, compared to the control group, the H-151 (STING inhibitor) group showed inferior fracture healing, while the SR-717 (STING agonist) group exhibited superior healing (**Figures 3A, B**). Further quantitative analysis of mouse bone structural parameters substantiated this effect. Relative to the control group, the bone tissue volume (BV), bone volume fraction (BV/TV), and trabecular bone indices such as bone mineral density (BMD) were lower in the H-151 group but higher in the SR-717 group. These findings suggest that the STING inhibitor H-151 delays fracture healing, whereas the STING agonist SR-717 promotes it (**Figures 3C, D**). Immunohistochemical analysis for STING revealed significant decreases in STING protein expression in the H-151 treated group and significant increases in the SR-717 group compared to the PBS control group, indicating successful drug intervention achieved differential regulatory effects on the cGAS-STING pathway during early fracture repair (**Figures 3E, H**). During healing, red cartilage tissue gradually transforms into bone tissue, facilitating fracture healing. Analysis of cartilage area through Safranin O-Fast Green staining showed significant inhibition of chondrocyte formation in the H-151 treated group, while SR-717 treatment promoted chondrocyte formation, consistent with Micro-CT observations (**Figures 3F, I**). Finally, TRAP staining, which presents osteoclast cytoplasm in purplish red against a blue background, showed that the STING inhibitor H-151 significantly suppressed osteoclasts, whereas the STING agonist SR-717 markedly increased them (**Figures 3G, J**). This confirms that activation of the cGAS-STING pathway can promote the generation of osteoclasts and further supports the pathway’s involvement in early fracture healing primarily through osteoclast generation.

### 3.4 *In vitro* experiments show that activation of the cGAS-STING pathway promotes RANKL-induced osteoclastogenesis and activation

**Figure 4:**
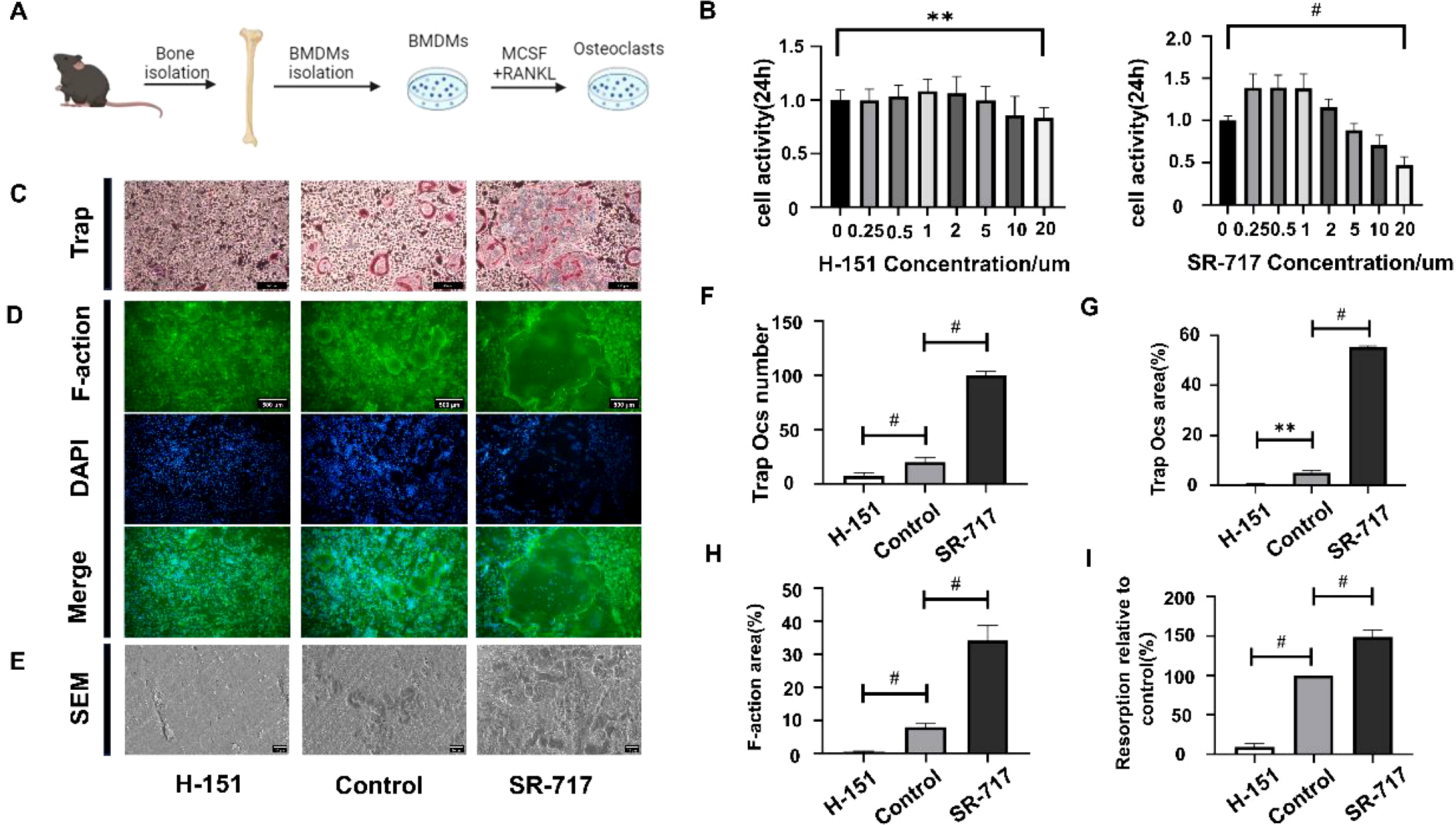
Activation of the cGAS-STING pathway promoted RANKL-induced osteoclast differentiation and resorption. **(A)** The process of inducing osteoclasts from mouse femoral bone marrow cells. **(B)** Evaluation of the cytotoxicity of RANKL-induced osteoclasts by adding different concentrations of H-151 and SR-717. **(C)** Detection of mature osteoclasts using TRAP staining. Scale bar 500 μm. **(D)** Fluorescent staining with DAPI (nuclei) and phalloidin labeled with fluorescein isothiocyanate (F-actin). Scale bar 500 μm. **(E)** Scanning electron microscope image capturing osteoclasts in bone resorption pits on bovine bone slices. Scale bar 30 μm. **(F, G)** Quantitative analysis of the number of TRAP-positive multinucleated cells and the percentage of osteoclast area. **(H)** Quantitative analysis of the percentage of the area occupied by F-actin rings. **(I)** Quantitative analysis of the percentage of bone resorption pit area. (n = 3 per group, statistical significance denoted by **P < 0.01, #P < 0.0001).

The experimental design is illustrated in the figure (**Figure 4A**). Cell viability was initially assessed using the CCK8 assay, choosing a concentration of 5um for H-151 as the STING inhibitor and 2um for SR-717 as the STING agonist (**Figures 4B, C**). Based on this, we explored the effect of the cGAS-STING pathway on RANKL-induced functional OCs formation *in vitro*. Analysis of TRAP-positive osteoclast numbers and areas post-TRAP staining revealed that STING inhibitor H-151 significantly inhibited the formation of multinucleated giant cells, whereas STING agonist SR-717 markedly promoted their formation compared to the control group (**Figures 4C, F, G**). Further experiments investigated the cGAS-STING pathway’s impact on osteoclast activity. It was observed that the F-actin ring area significantly decreased after H-151 intervention, indicating reduced osteoclast activity; conversely, SR-717 intervention significantly increased the F-actin ring area, indicating enhanced osteoclast activity (**Figures 4D, H**). Lastly, the effect of the cGAS-STING pathway on osteoclast bone resorption function was assessed using an *in vitro* bone pit assay. Using bovine bone slices as a standardized and controllable bone environment facilitated the exploration of mature osteoclasts’ bone resorption function. Compared to the control group, H-151 intervention significantly reduced the number and area of bone pits, whereas SR-717 intervention significantly increased them (**Figures 4E, I**).

### 3.5 Activation of the cGAS-STING pathway promotes RANKL-induced osteoclast differentiation and the NF-κB signaling pathway

**Figure 5:**
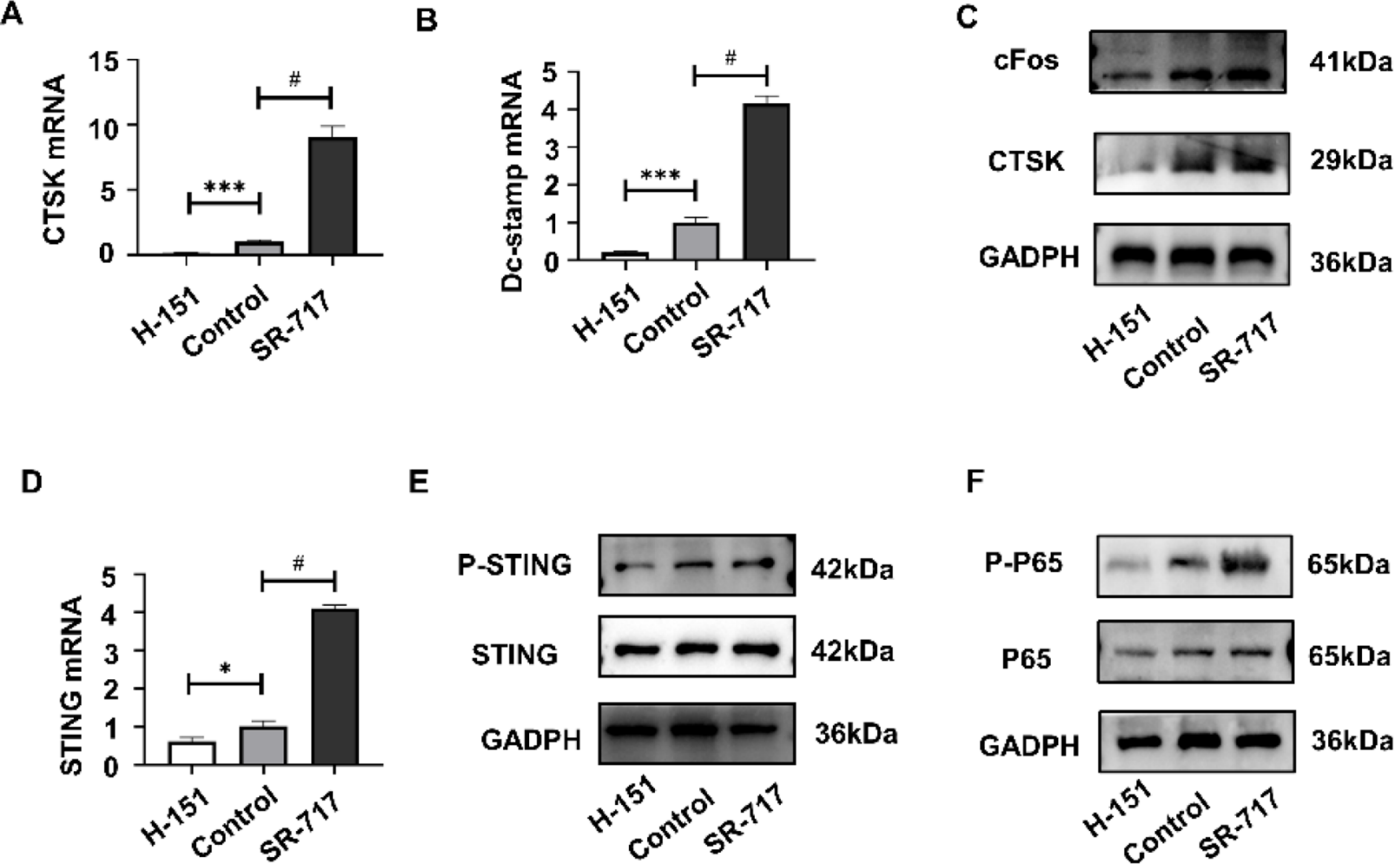
Activation of the cGAS-STING pathway promotes RANKL-induced osteoclast differentiation and the NF-κB signaling pathway. **(A, B)** Real-time quantitative PCR was used to detect the mRNA expression levels of Cathepsin K (Ctsk) and Dendritic Cell-Specific Transmembrane Protein (Dc-stamp). **(C)** Western blotting quantified the protein expression levels of CTSK and cFos. **(D, E)** The expression levels of STING mRNA and protein were assessed using qPCR and Western blotting. **(F)** Western blotting was employed to examine the expression and phosphorylation levels of the P65 protein. Statistical significance: *P < 0.05, ***P < 0.001, #P < 0.0001.

To further investigate the molecular mechanisms and signaling pathways underlying the regulatory effects of intervening in the cGAS-STING pathway on osteoclast differentiation, we examined the expression levels of osteoclast-specific messenger RNAs (cFos, DC-STAMP). Compared to the control group, these specific messenger RNAs exhibited varying degrees of decrease under H-151 intervention and varying degrees of increase under SR-717 intervention (**Figures 5A, B**). Additionally, the expression levels of osteoclast characteristic proteins (CTSK and c-Fos) were assessed using Western Blot, confirming a reduction in CTSK and c-Fos protein expression following H-151 intervention and an increase following SR-717 intervention, aligning perfectly with qRT-PCR results (**Figure 5C**).

Lastly, a Western Blot analysis of the key molecule (p65) protein in the NF-κB signaling pathway, critical for osteoclast differentiation, was performed. Results showed no difference in the total amount of the key NF-κB signaling molecule (p65) protein across different groups. However, phospho-protein (p-p65) expression significantly decreased under H-151 intervention and significantly increased under SR-717 intervention (**Figures 5D–F**). This indicates that regulation of the cGAS-STING pathway affects the phosphorylation status of the NF-κB signaling pathway, potentially representing one of the key mechanisms leading to changes in osteoclast differentiation and activity.

## 4 Discussion

Recent studies have highlighted a connection between the cGAS-STING pathway and the onset and progression of orthopedic diseases, such as osteoarthritis, spinal cord injury, and osteolysis (17–20). Additionally, research has discovered a significant role for the STING signaling pathway in bone formation, particularly in the context of autoimmune diseases, where deficiencies in STING significantly inhibit bone accumulation (21). This suggests the cGAS-STING pathway might also play a role in the fracture healing process. Initially, we prepared a fracture model in mice and performed transcriptomic sequencing analysis on callus tissues on days 7 and 21 post-surgery, identifying significant differences in the cGAS-STING pathway between early and late stages of fracture healing. Analysis revealed a higher expression of STING on day 7 than on day 21, suggesting that cGAS-STING activation primarily occurs in the early phase of fracture healing. This early activation may relate to the acute inflammatory response following fracture trauma. Further immunohistochemical analysis of STING at different post-surgery time points (days 3, 7, and 14) showed almost no STING expression in normal, untreated bone tissue, indicating very low levels of STING expression in healthy bone tissue. However, post-fracture model preparation, we noticed the appearance of STING-positive regions, suggesting the cGAS-STING pathway is stimulated by the fracture and participates in the healing process. Following a fracture, damaged bone and surrounding soft tissues rapidly initiate an inflammatory response, releasing a plethora of cytokines and chemokines that attract immune cells to the injury site (13, 22). The activation of the cGAS-STING pathway may be part of this response, aiding in the stimulation and regulation of the immune response to promote the clearance of the wound and early tissue rebuilding. By day 14 post-surgery, the decrease in STING expression levels reflects a possible transition from the inflammatory phase to the repair and rebuilding stages of fracture healing. At this stage, the inflammatory response diminishes, callus matures, and bone tissue begins to reconstruct and remodel (23, 24). The reduced activity of the cGAS-STING pathway may be due to decreased sensitivity to DNA damage and inflammation in the later stages of healing, with the body’s repair mechanisms beginning to dominate the healing process. This dynamic change suggests the regulation of the cGAS-STING pathway in fracture healing could be a potential therapeutic target.

To further understand the role of the cGAS-STING pathway in fracture healing, we conducted *in vivo* experiments using the STING inhibitor H-151 and the STING agonist SR-717. H-151 acts primarily as an antagonist of STING (stimulator of interferon genes), where palmitoylation of Cys91 is a key step in STING activation. H-151 inhibits activation by covalently binding to a specific site on the STING protein (Cys91) (25, 26). SR-717, a non-nucleotide STING agonist, works by mimicking the structure of STING’s natural agonist cGAMP, directly binding to STING, thus promoting its activation (27). The use of STING inhibitor H-151 and STING agonist SR-717 allowed us to simulate the regulatory effects of the cGAS-STING pathway in the early stages of fracture healing. Our results show that the cGAS-STING pathway not only primarily acts on osteoclasts but is also closely related to the NF-κB signaling pathway. To delve deeper into the impact of the cGAS-STING pathway on fracture healing, we quantitatively analyzed the bone structural parameters of mice using microCT on days 14 and 28 post-surgery. Compared to the PBS control group, we observed significant delays in fracture healing in the H-151 treated group, whereas fracture healing was notably faster in the SR-717 treated group. Furthermore, Safranin O-Fast Green staining analysis of the cartilage area revealed significant inhibition of chondrocyte formation post-H-151 treatment and significant promotion post-SR-717 treatment, reaffirming the promotive role of cGAS-STING pathway activation in mouse fracture healing. Lastly, TRAP staining showed that compared to the PBS control group, H-151 treatment significantly inhibited osteoclast generation, whereas SR-717 treatment significantly promoted it, further supporting the involvement of the cGAS-STING pathway in fracture healing primarily through mediating osteoclast generation.

Osteoclasts play a crucial role in the fracture healing process, primarily responsible for the resorption and degradation of bone tissue, creating the necessary space for new bone regeneration. The generation and differentiation of osteoclasts are regulated by the cytokines Macrophage Colony-Stimulating Factor (M-CSF) and NF-κB ligand receptor activator (RANKL). M-CSF drives monocyte proliferation and provides a macrophage fate determination signal while acting as a survival factor. RANKL drives differentiation, fusion, and further development into functional osteoclasts (28–30). Mature osteoclasts possess various morphological and functional characteristics, with TRAP, an enzyme secreted by osteoclasts during bone resorption, playing a key role (31, 32). TRAP aids in acidifying the bone surface, a necessary step for bone resorption (33). Compared to the control group, intervention with the STING inhibitor H-151 significantly inhibited the formation of multinucleated giant cells, whereas the STING agonist SR-717 intervention markedly promoted their formation. Mature multinucleated osteoclasts can form F-actin rings, one of the most iconic features of osteoclast differentiation (34). Compared to the control group, the number and area of F-actin rings significantly decreased post-H-151 intervention, indicating weakened osteoclast activity; conversely, SR-717 intervention significantly increased the number and area of F-actin rings, enhancing osteoclast activity. We also used bovine bone slices to validate the differences in mature osteoclast bone resorption function caused by intervening in the cGAS-STING pathway, observing a significant reduction in the number and area of bone pits post-H-151 intervention, whereas SR-717 intervention significantly increased them. This further confirms that activation of the cGAS-STING pathway promotes the bone resorption function of mature osteoclasts. Additionally, during the RANKL-induced differentiation of BMMs into osteoclasts, numerous osteoclast genes are expressed, their activity and expression levels determining the bone resorption capacity of osteoclasts. Activated T-cell nuclear factor c1 (NFATc1), a transcription factor, is a key regulator of osteoclast differentiation. NFATc1 is activated upon stimulation of osteoclast precursors by RANKL (nuclear factor κB ligand) and migrates to the nucleus to promote the expression of osteoclast-specific genes, including a variety of genes such as Tartrate-Resistant Acid Phosphatase (TRAP) and DC-STAMP (35, 36). CTSK, a lysosomal enzyme that operates in acidic environments, is one of the key enzymes osteoclasts use to degrade bone matrix (37). DC-STAMP, a cell surface protein, plays a crucial role in the multinucleation process of osteoclasts. c-Fos, one of the upstream regulators of NFATc1, works in conjunction with NFATc1 to promote osteoclast differentiation and activity (38). Molecular experiments have proven that activation of the cGAS-STING pathway promotes RANKL-mediated osteoclast generation and activation. Phosphorylation of the p65 subunit is a key step in the activation of the NF-κB signaling pathway (39), and we have also verified that post-cGAS-STING pathway treatment is related to the key pathway NF-kB signaling pathway involved in osteoclast formation.

We acknowledge that the mechanistic conclusions of this study rely primarily on pharmacologic modulation using H-151 and SR-717, and that these agents, while well-characterized, may have off-target effects. To mitigate this concern, we employed a multi-pronged approach combining transcriptomic analysis, immunohistochemistry, and *in vitro* validation, all of which yielded consistent results. The convergent evidence from both inhibitor and activator studies—showing opposite effects on osteoclastogenesis and fracture healing—further supports the specificity of our observations. Nevertheless, we recognize that genetic models such as STING knockout mice would provide more definitive causal evidence. Future studies incorporating STING-deficient animals will be valuable to validate and extend our findings.

Recent advances have established the cGAS-STING pathway as a critical regulator of skeletal homeostasis and pathology. A comprehensive review has systematically summarized its involvement in orthopedic conditions including osteoporosis, osteoarthritis, and delayed fracture healing, highlighting its central role in mediating sterile inflammation and bone remodeling (40). In the context of osteoclast biology, Guo et al. demonstrated that activated NETosis of bone marrow neutrophils promotes osteoclastogenesis via the cGAS-STING/AKT2 pathway, contributing to osteoporosis in ovariectomized mice (41). This finding directly supports our observation that STING activation enhances osteoclast differentiation and activity. Conversely, Song et al. discussed the role of STING signaling in inflammaging, suggesting that chronic STING activation may contribute to age-related bone loss (20). Regarding bone formation, Qiu et al. recently reported that RAD51 promotes osteogenic differentiation by suppressing cGAS-STING signaling, indicating that this pathway negatively regulates osteoblast differentiation. Furthermore, Li et al. demonstrated that TRIM30a coordinates neutrophil-macrophage crosstalk to resolve inflammation and drive osseointegration via suppressing the NETosis/cGAS-STING axis. Our findings extend this body of work by demonstrating, for the first time, the temporal dynamics of STING activation during fracture healing. Unlike chronic inflammatory conditions where sustained STING activation drives pathological bone loss, we observed that STING is transiently activated during the early inflammatory phase of fracture repair (days 3–7 post-fracture) and that this acute activation promotes osteoclast differentiation and accelerates bone healing. This pro-reparative role contrasts with the detrimental effects of STING in diseases such as osteoporosis and highlights the critical importance of temporal and microenvironmental factors in determining pathway outcomes. The convergence of evidence from both inhibitor and activator studies in our work further supports the specificity of STING-mediated regulation in this context.

In this study, the transcriptomic comparison was performed between callus tissues harvested at early (day 7) and late (day 21) stages of fracture healing, without including non−fractured bone as a baseline control. While this design allowed us to capture stage−specific gene expression dynamics during repair, it did not provide a direct comparison to the uninjured state. Nevertheless, immunohistochemical analysis revealed negligible STING expression in normal bone (**Figure 1C**), suggesting that the cGAS−STING pathway is largely inactive under basal conditions. Future investigations should incorporate uninjured bone tissue as a transcriptomic reference to more precisely delineate the activation kinetics and regulatory networks of the cGAS−STING pathway throughout fracture healing.

**Figure 6:**
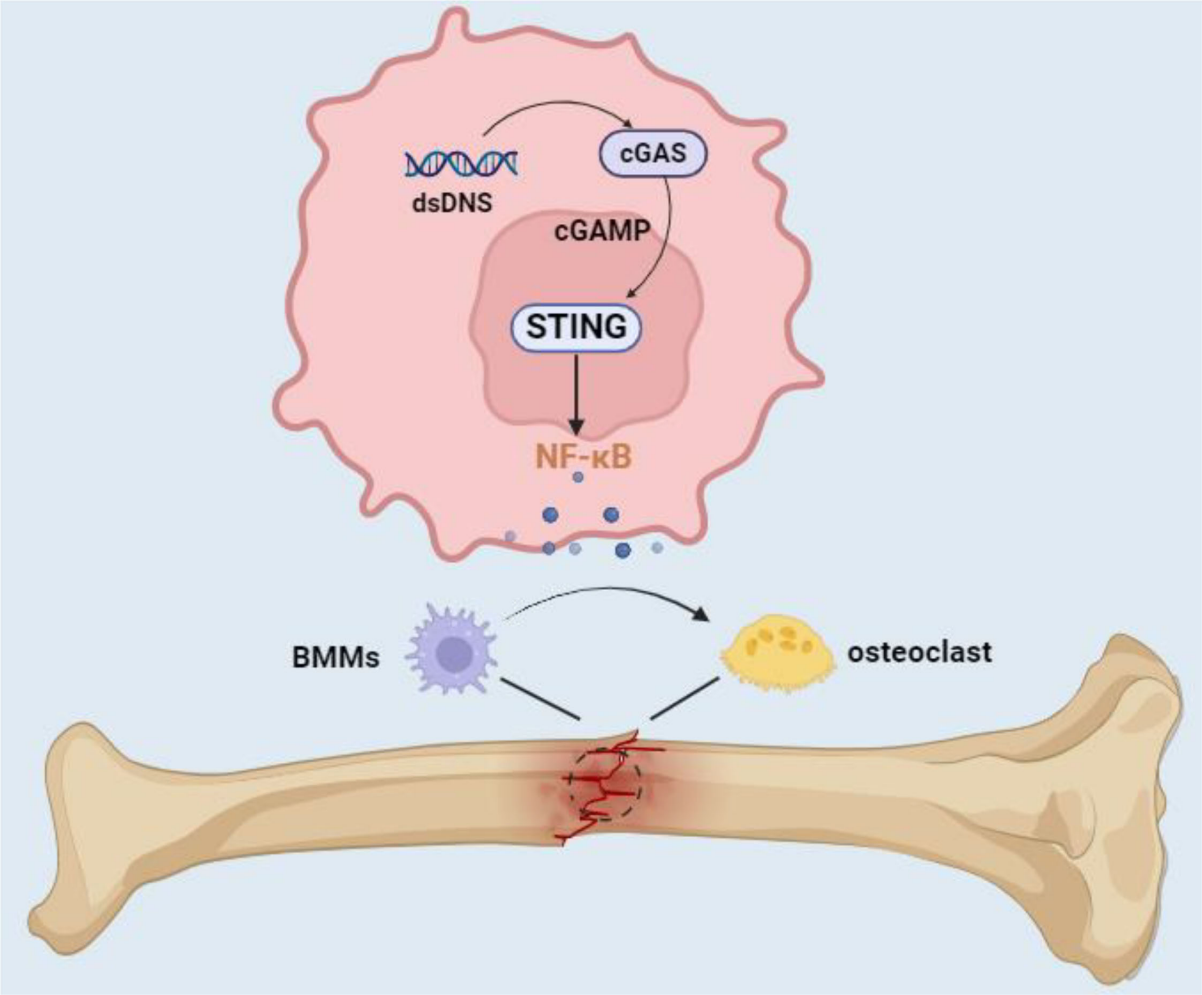
This study provided a schematic of the potential role of the cGAS-STING pathway in early fracture healing.

In summary, our study identifies a previously unrecognized role of the cGAS-STING pathway in the early inflammatory phase of fracture healing, where its activation promotes osteoclastogenesis and accelerates bone repair. These findings not only extend the current understanding of cGAS-STING function beyond pathological inflammation but also highlight its therapeutic potential in bone regeneration by targeting stage-specific mechanisms (**Figure 6**). This provides significant evidence for the development of related therapeutic drugs, offering theoretical bases and clinical guidance for promoting fracture healing and the development of new treatment strategies.

## Data Availability

RNA-seq data are available at GEO accession numbers GSE308052. All data generated or analyzed during this study are included in this published article. The more detailed data that support the findings of this study are available on request from the corresponding author upon reasonable request.
